# Cardiovascular disease, risk factors and heart rate variability in the elderly general population: Design and objectives of the CARdiovascular disease, Living and Ageing in Halle (CARLA) Study

**DOI:** 10.1186/1471-2261-5-33

**Published:** 2005-11-11

**Authors:** Karin H Greiser, Alexander Kluttig, Barbara Schumann, Jan A Kors, Cees A Swenne, Oliver Kuss, Karl Werdan, Johannes Haerting

**Affiliations:** 1 Institute of Medical Epidemiology, Biostatistics and Informatics, Martin-Luther-University Halle-Wittenberg, Magdeburger Str. 27, 06097 Halle (Saale), Germany; 2 Department of Medical Informatics, Erasmus Medical Center Rotterdam, PO Box 1738, Rotterdam, The Netherlands; 3 Department of Cardiology, Leiden University Medical Center, PO Box 9600, 2300 RC Leiden, The Netherlands; 4 Department of Medicine III, Martin-Luther-University Halle-Wittenberg, Ernst-Grube-Str. 40, 06097 Halle (Saale), Germany

## Abstract

**Background:**

The increasing burden of cardiovascular diseases (CVD) in the ageing population of industrialized nations requires an intensive search for means of reducing this epidemic. In order to improve prevention, detection, therapy and prognosis of cardiovascular diseases on the population level in Eastern Germany, it is necessary to examine reasons for the East-West gradient of CVD morbidity and mortality, potential causal mechanisms and prognostic factors in the elderly.

Psychosocial and nutritional factors have previously been discussed as possible causes for the unexplained part of the East-West gradient. A reduced heart rate variability appears to be associated with cardiovascular disease as well as with psychosocial and other cardiovascular risk factors and decreases with age. Nevertheless, there is a lack of population-based data to examine the role of heart rate variability and its interaction with psychosocial and nutritional factors regarding the effect on cardiovascular disease in the ageing population. There also is a paucity of epidemiological data describing the health situation in Eastern Germany. Therefore, we conduct a population-based study to examine the distribution of CVD, heart rate variability and CVD risk factors and their associations in an elderly East German population. This paper describes the design and objectives of the CARLA Study.

**Methods/design:**

For this study, a random sample of 45–80 year-old inhabitants of the city of Halle (Saale) in Eastern Germany was drawn from the population registry. By the end of the baseline examination (2002–2005), 1750 study participants will have been examined. A multi-step recruitment strategy aims at achieving a 70 % response rate.

Detailed information is collected on own and family medical history, socioeconomic, psychosocial, behavioural and biomedical factors. Medical examinations include anthropometric measures, blood pressure of arm and ankle, a 10-second and a 20-minute electrocardiogram, a general physical examination, an echocardiogram, and laboratory analyses of venous blood samples. On 200 participants, a 24-hour electrocardiogram is recorded. A detailed system of quality control ensures high data quality. A follow-up examination is planned.

**Discussion:**

This study will help to elucidate pathways to CVD involving autonomic dysfunction and lifestyle factors which might be responsible for the CVD epidemic in some populations.

## Background

Cardiovascular diseases (CVD) are the leading cause of death and morbidity in industrialized nations, accounting for about 50% of all deaths [1]. In old age, both incidence and prevalence of CVD are increasing. Although the mortality of coronary heart disease has decreased since the 1970s, the prevalence, incidence and mortality of chronic heart failure has persisted or even increased [2-5]. In the ageing population, chronic heart failure (CHF) is an increasing public health problem and poses a mounting economic burden on societies and their health care systems due to its association with frequent hospitalizations and the need for long-term pharmacological treatment [2,6-9].

Moreover, in spite of the general decline of cardiovascular mortality over the past decades, an increasing East-West gradient within Europe has been observed with decreasing life-expectancy and higher rates of cardiovascular morbidity and mortality in Eastern European countries which seems to arise from premature CVD [10,11]. This discrepancy has grown since the political changes and socioeconomic discontinuities in Eastern Europe at the beginning of the 1990s and cannot be explained entirely by the classical risk factors for cardiovascular diseases alone [12]. Even within Germany, an East-West gradient of CVD mortality is still present after re-unification [13]. However, there is a lack of population-based data on CVD morbidity and risk factors to further analyse the extent, cause and potential for reduction of the increased CVD burden in the East. Among the newer risk factors discussed as potential explanations for the East-West gradient are nutritional factors, alcohol and psychosocial factors [10,14,15]. Psychosocial factors such as depression, social isolation, job strain and hostility are associated with higher CVD risk [16-19] and are differentially distributed by socioeconomic status [20-23].

Autonomic dysfunction as indicated by a reduced heart rate variability (HRV) is increasing with age [24,25] and has been observed to be associated with an increased risk for incident coronary heart disease (CHD), CVD mortality [26-30] and worse prognosis in patients with CHD or heart failure [31-34]. However, there is growing evidence that adverse psychosocial factors might also be associated with a reduced HRV and other measures of sympathovagal imbalance [35-38]. A reduced heart rate variability appears to integrate negative factors such as disease and stress. Thus, autonomic dysfunction, of which HRV is a marker, might play an important role in mediating the effect of social inequality, psychosocial factors and other cardiovascular risk factors on CVD causation and prognosis [39-42].

For inflammatory factors such as C-reactive protein (CRP) and cytokines (tumour necrosis factors, interleukin-6), higher levels have been observed in elderly subjects and in subjects at higher risk of chronic heart failure (CHF) [43,44]. They have also been associated with a reduced HRV [45]. However, the role of inflammatory factors and their interaction with HRV in the pathogenesis of heart failure is still not well understood. There is a lack of population-based studies to investigate the potential mechanisms involving HRV, inflammatory factors and psychosocial factors underlying causation and prognosis of CHF.

The examination of heart rate variability in a general population might provide valuable information to elucidate some of the pathways relevant for the development and prognosis of CVD. It might also help to explain the mechanisms underlying the observed associations of psychosocial factors with CVD. Moreover, unfavourable psychosocial factors and a reduced heart rate variability could be among the factors responsible for the higher CVD mortality in Eastern European and Eastern German populations as compared to their Western counterparts. The examination of reasons for regional variation such as the East-West gradient of CVD morbidity and mortality as well as of potential mechanisms of causation and of prognostic factors in the elderly could lead to advances in the prevention, detection, therapy and prognosis of cardiovascular diseases on the population level.

Furthermore, the role of HRV as a potential additional screening tool for the identification of subjects at high risk of acquiring cardiovascular diseases or of suffering fatal adverse events should be explored. However, there is a lack of population-based data regarding the distribution of HRV and its interaction with other CVD risk factors in the general ageing population. The recording of 24-hour holter electrocardiograms (ECG) or ECGs of at least 2 hours duration is still the recommended standard for HRV analysis in clinical practice [46]. While 24-hour ECGs record the dynamic component of HRV derived from fluctuations in daily activities and thus contain other information than short-term ECGs, a great advantage of short ECG recordings in resting state is that they can be highly standardized and are easier to apply in larger populations. For screening purposes in a general population, the recording of long-term ECGs is impractical.

Although short-term and long-term ECGs measure different components of HRV, one study in healthy young men showed a high predictive value of short-term ECGs for long-term ECGs [47]. Unfortunately, there is a lack of studies assessing the validity and predictive value of short-term ECGs for the determination of HRV as compared to HRV measures derived from 24-hour ECGs in the general, elderly population. Therefore, it is important to examine whether ECGs of shorter recording duration (e. g., 20 minutes) are a valid basis for the identification of subjects with reduced HRV in the general population.

This paper presents the design and objectives of the **CAR**diovascular disease, **L**iving and **A**geing in Halle (CARLA) Study.

The aims of this study are:

1. to examine the distribution of different parameters of heart rate variability and the prevalence of a reduced HRV in a representative sample of an elderly East German population;

2. to examine the prevalence of cardiovascular diseases and their association with HRV, psychosocial and socioeconomic factors, and inflammatory and classical cardiovascular risk factors in an ageing general population;

3. to identify factors responsible for the epidemic of chronic heart failure in the ageing population, and to identify potential areas of prevention;

4. to elucidate reasons for regional variations in CVD morbidity and mortality; and

5. to assess the potential of HRV derived from short-term ECGs as a screening tool in the general population for the detection of subjects at high risk of CHD or CHF and for risk stratification in chronic heart failure or other cardiovascular diseases.

A planned follow-up study will allow the determination of incident events.

## Methods/design

The design of the CARLA study is a population-based epidemiologic cross-sectional study with the aim of a prospective follow-up of the examined study subjects, thus leading to a cohort study. The study was approved by the Ethics Committee of the Medical Faculty of the Martin-Luther-University Halle-Wittenberg and by the State Data Privacy Commissioner of Saxony-Anhalt. The study is conducted by the Institute of Medical Epidemiology, Biostatistics and Informatics in cooperation with the Department of Internal Medicine III (Cardiology, Angiology and Medical Intensive Care) at the Martin-Luther-University Halle-Wittenberg.

### Study population, sampling and recruitment procedure

The base population for the study participants are male and female inhabitants of German nationality of the city of Halle (Saale) in Saxony-Anhalt, Eastern Germany, aged 45 to 80 years. The city of Halle has approximately 240 000 inhabitants and is the largest city in Saxony-Anhalt. A random sample of 5000 men and women aged 45 to 80 years at the time of the sampling (July 2002) was drawn from the population registry of the city of Halle.

The sampling was done within the age strata 45–49, 50–54, 55–59, 60–64, 65–69, 70–74 and 75–80 years. In all but the last stratum, equal numbers of men and women were drawn (322 women and 323 men per stratum). The age stratum 75–80 years was oversampled with twice as many inhabitants drawn into this sample than for each of the other strata (625 women and 625 men). This was done in order to account for lower expected response rates in the oldest group.

The recruitment of study subjects began in December 2002 and is aimed to be completed by the end of the year 2005. When completed, the final study population will comprise 1750 persons. Up to July 2005, about 1500 subjects were examined. Assuming comparable response rates within all age-sex strata except the oldest one, we expect to recruit about 120 men and 120 women in each 5-year-age group from 45 to 74 years, and 155 men and women each in the age-group 75 to 80 years. An overall response rate of 70 % is aimed at.

The recruitment of study subjects has been done by inviting consecutive waves of random sub-samples of the original population sample. Therefore, not all persons originally drawn from the population registry have to be invited in order to obtain a representative sample of the Halle population aged 45 to 80 years.

The method of recruitment applied in the CARLA study was selected after discussion with experts about the most efficient strategies to achieve the desired response rate. The recruitment strategy includes at least two written invitations and active contact attempts by the study personnel via telephone or home visit if the eligible person does not respond to the written invitation. The mailed invitation contains a letter of invitation, a detailed description of the aims and examination procedures of the study, and a copy of the approval letter of the Data Privacy Commissioner for Saxony-Anhalt. Different recruitment strategies are followed for subjects for whom a home telephone number can be identified from the official telephone directories and for those with an ex-directory or no telephone number:

Persons with known telephone numbers are identified and receive a letter of invitation announcing a subsequent phone call by the study personnel. If the person does not respond actively to the invitation letter, study personnel make at least 10 attempts to establish telephone contact at different times of the day and on different days. If no contact can be made over at least six weeks, a reminder invitation is mailed to the subject, offering the option of replying with a prepared reply-paid card indicating convenient examination dates. If the second invitation does not lead to a contact with the subject or to verification of his response state (e. g. moved with unknown address, too ill to participate in the study, deceased, or unwilling to participate in the study), a home visit is performed by study personnel. Several attempts are made to localize the study subject or to retrieve information from relatives or neighbours regarding the subject's correct address of residence or telephone number, or their willingness or ability to participate.

For those subjects without a registered telephone number, two invitation letters with reply-paid cards are sent before entering the home visit phase.

**Figure 1:**
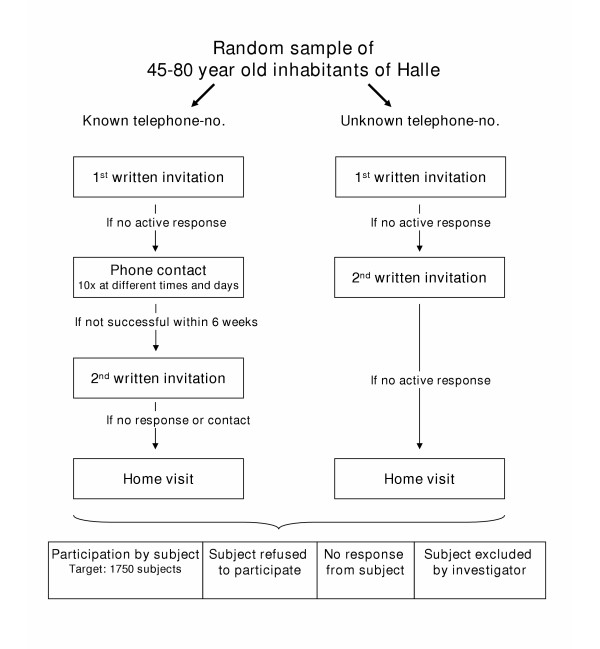
Recruitment scheme for CARLA baseline examination.

All direct contact attempts (phone or home visit) are made by trained and certified study personnel and are documented in detail. At each home visit which does not result in contact with the invited subject or another member of the household, the home visitor leaves a pre-printed note indicating that a visit has been made and indicating how contact can be made with the researchers. Figure 1 shows the recruitment scheme of the CARLA Study.

A letter confirming the date of examination is sent to subjects who agreed to participate in the study and contains a consent form, a map of the study centre and a self-administered questionnaire.

### Data collection – interview, questionnaires and examination procedures

In order to provide a basis for pooled data analyses and to permit valid comparisons with other study regions and populations, the CARLA Study deliberately uses highly standardized and validated instruments of data collection which have been applied in several completed or ongoing national and international cardiovascular disease epidemiologic studies. Questionnaire items and examination procedures were selected and adapted from i.) the KORA Study Augsburg in Bavaria, Southern Germany (the continuation of the MONICA Surveys in Augsburg) [48]; ii.) the SHIP Study Greifswald in Pomerania, North-Eastern Germany [49]; iii.) the EPIC Potsdam Study, Eastern Germany [50]; iv.) the Rotterdam Study, The Netherlands [51]; and v.) the HAPIEE Study which includes study regions in the Czech Republic, Poland, Russia and Lithuania [52].

After selection of interview items and examination procedures, a pretest was performed to test instruments and logistics of data collection. Slight modifications were made before recruitment of the study subjects started.

The data collection consists of a standardized, computer-assisted personal face-to-face interview, a self-administered questionnaire, a medical examination by a trained and certified study nurse, and a physical examination and a transthoracic echocardiogram performed by a physician who has been specifically certified for this study. The examination takes place at the CARLA study centre in the University Hospital of the Martin-Luther-University Halle-Wittenberg. The average duration is 3.5 hours per subject.

### Interview

The computer-assisted interview was programmed with the DAIMON interview programme which was also used in the KORA 2000 Survey Augsburg [53]. The interview collects information on sociodemographic and socioeconomic data, medical history and cardiovascular risk factors. In detail, it includes questions on household income, educational level and occupation of the interviewee, and his or her parents and partner (where appropriate), material circumstances, psychosocial factors such as social support, unemployment and job security, changes in socioeconomic factors and social relations since the German re-unification in 1990, utilization of health care services, family medical history of chronic diseases, and history of any physician-diagnosed cardiovascular diseases (coronary heart disease, stroke, arterial hypertension), diabetes, hypercholesterolemia, thyroid disease, osteoporosis, chronic bronchitis, rheumatoid arthritis and cancer. It furthermore contains the Rose questionnaire on angina pectoris and intermittent claudication [54] and information on lifestyle-factors such as smoking, diet, alcohol consumption and physical activity. The interview lasts approximately one hour. Details of the sources of the interview modules are listed in table 1. Information on the use of medication during the seven days preceding the examination is collected by the study nurse with the computer-based IDOM programme [55]. It integrates the GKV medication database, the official medication database of the Wissenschaftliches Institut der Ortskrankenkassen (WIdO, Scientific Institute of the Local Sickness Funds) which is continuously updated and allows an automated search for individual drugs by PZN number (central pharmanumber) and direct retrieval of ATC codes [56].

**Table 1:** Components and sources of the interview used in the baseline survey of the CARLA Study

**Topics of interview module**	**Source (reference, original study)**
Sociodemographic factors: 1. Subject's own education, occupational status, net household income 2. Parental education and occupational status 3. Partner's education and occupational status	1. Demographic standards for Germany [81] 2. adapted according to [81] 3. adapted according to [81]
Utilization of medical services	Modified from SHIP [49,82]
Chronic diseases: own medical history 1. cardiovascular diseases: coronary heart disease, stroke, hypertension 2. chest pain 3. intermittent claudication 4. hypercholesterolemia 5. diabetes 6. thyroid disease 7. osteoporosis 8. rheumatoid arthritis 9. chronic diseases 10. cancer Chronic diseases: family history 1. myocardial infarction, stroke, hypertension 2. diabetes 3. cancer Menopausal state and use of hormone replacement therapy	All adopted from SHIP [49,82] and KORA/MONICA [48,83-85]; Rose questionnaire for chest pain and intermittent claudication [54]
Medication use during the preceding 7 days	IDOM software developed by GSF, based on the GKV medication database for Germany, used in KORA study [55,56]
Health related behaviour/lifestyle factors 1. diet/nutritional habits – short qualitative food frequency list 2. alcohol use 3. physical activity 4. smoking	1. adopted from KORA/MONICA [83,86] 2. adopted from HAPIEE [52] 3. Baecke questionnaire [87], also used in SHIP and ARIC 4. adapted and shortened from smoking questionnaire developed by W. Ahrens, BIPS
Social support	Modified version of Berkman-questionnaire (translation by J. Siegrist used in KORA/MONICA) [48,84,88,89]
Unemployment, job insecurity	Adopted from SHIP [49,82] and HAPIEE [52]
Material circumstances	Adopted from HAPIEE [52]

ARIC = Atherosclerosis Risk in Communities; BIPS = Bremen Institute for Prevention Research and Social Medicine; CES-D = Center of Epidemiological Studies Depression Scale; EPIC = European Prospective Investigation into Cancer and Nutrition; HAPIEE = Health, Alcohol and Psychosocial Factors in Eastern Europe; KORA = Cooperative Health Research in the Augsburg Region; MONICA = Monitoring Trends and Determinants in Cardiovascular Disease; SF 12 = Short Form Health Survey Questionnaire; SHIP = Study of Health in Pomerania

### Self-administered questionnaire

The self-administered questionnaire contains the food-frequency questionnaire (FFQ) also used in the EPIC Potsdam Study [50] follow-up, quantitative questions about alcohol drinking patterns aimed at obtaining information on binge drinking, questions about health beliefs, social networks, the CES-D questionnaire on depression, the SF-12 questionnaire on self-rated health and health-related quality of life, the effort-reward imbalance questionnaire developed by Siegrist [57,58], and questions about security in the neighbourhood environment before and after the re-unification in 1990. The EPIC FFQ is usually completed at home after the examination date and sent back via reply-paid envelope.

Details on the sources of the questionnaire modules are listed in table 2.

**Table 2:** Components and sources of the self-administered questionnaire used in the baseline survey of the CARLA Study

**Topics of questionnaire modules**	**Source (reference, original study)**
Health related behaviour/lifestyle factors 1. diet/nutritional habits – quantitative food frequency questionnaire (FFQ) 2. alcohol use – binge drinking	1. adopted from EPIC [50] 2. adopted from HAPIEE [52]
Social networks	Modified version of Berkman-questionnaire (translation by J. Siegrist used in KORA/MONICA) [48,84,88,89]
Perceived security in neighbourhood environments before and after German re-unification in 1990	adopted and translated from HAPIEE [52,90-92]; similar questions in SHIP [82]
Health beliefs	Adopted from HAPIEE [52]
Health-related quality of life	Social functioning questionnaire SF 12 [93]
Job strain, effort-reward imbalance	Effort-reward imbalance questionnaire by J. Siegrist [57,58]
Depression scale	German translation of the CES-D depression scale by Kohlmann & Gerbershagen [94-96]

Abbreviations: same as in table 1

### Examination procedures

The medical examination includes the measurement of anthropometric parameters and arterial blood pressure, the determination of the ankle-arm index of systolic blood pressure, the recording of a 10-second- and a 20-minute electrocardiogram, a trans-thoracic echocardiogram, and the drawing of a venous blood sample.

At the beginning of the examination, the subject is seated comfortably in a chair and asked to assume the position required later for the correct measurement of sitting blood pressure. Then, the medication currently taken by the study subject is recorded using the IDOM programme (see table 1). After a resting period of at least five minutes, the measurement of systolic and diastolic blood pressure is started. Blood pressure is measured with the OMRON HEM-705CP automated oscillometric blood pressure device [59] according to the procedure employed in the SHIP and KORA/MONICA Study [48,49,55]. The OMRON HEM-705CP device meets the criteria defined for the use in clinical trials by the Association for the Advancement of Medical Instrumentation and the British Hypertension Society. The size of the cuff is selected according to the arm circumference (circumference <32 cm: normal adult cuff, circumference 32 – <42 cm: large adult cuff). Three measurements are performed on the left arm with a three-minute delay between each pair of measurements. Heart rate is counted manually during the resting period. Data entry of the measurements is done immediately using the DAIMON screen developed by the GSF which includes a timer for the three-minute delay between the blood pressure measurements [53].

The anthropometric measurements follow the procedures used in the MONICA/KORA and SHIP study [48,49,55]. Weight and height are measured with the SECA 701 digital scale and the SECA 220 height measuring system. Waist and hip circumference are measured using a flexible tape, with the study subject standing in front of a full-size mirror which allows checking the horizontal position of the tape. Weight is recorded with a precision of 100 g, and height, waist and hip circumference to the nearest 0.1 cm.

The individual is then asked to lie down and rest for at least five minutes before the measurement of supine systolic blood pressure at arm and ankle is commenced for the determination of the ankle-arm index as indicator of peripheral arterial disease (PAD). The protocol for the measurement of ankle-arm index using the OMRON HEM-705CP both at arm and leg was developed by the CARLA Study. First, a simultaneous measurement of blood pressure (BP) at both arms is performed. For the BP measurements used for the calculation of ankle-arm index, the OMRON HEM-705CP device remains on the arm with the higher systolic blood pressure. The circumference of both calves is determined at the midpoint of the BP cuff to select the adequate cuff size. The cuff is positioned approximately 5 cm above the inner ankle over the posterior tibial artery in contour wrap technique [60]. Measurement of BP is started simultaneously on arm and ankle, using two OMRON HEM-705CP devices at the same time. First, two measurements are performed at the right ankle, followed by two measurements at the left ankle. Between each pair of measurements, there is a one-minute delay.

Following the supine BP measurement two resting electrocardiograms (ECG) are recorded: one 10-second and one 20-minute 12-lead ECG. The study subject remains in supine position and is not allowed to raise from the beginning of the supine BP measurements until completion of the ECGs. The attachment of the electrodes follows the standard procedures for measuring and marking of the electrode positions using a DAL-square as described in the ARIC Manual 5 Electrocardiography [61] and adopted by the KORA/MONICA Study [55]. The ECGs are recorded using a Cardio Control Medical Diagnostic Workstation 1.3.1 with a Cardio Perfect MD Recorder with a sampling rate of 600 Hz (Welch Allyn Cardio Control, Delft, NL) and stored digitally. The study subject is asked to remain in supine position and not to speak throughout the ECG recording. The 10-second ECG is recorded first, and a printout is provided for interpretation by the study physician.

It is required that the study subject has remained in supine resting position for at least 20 minutes before the recording of the 20-minute ECG is started. The baroreceptors which sense the blood pressure in the aorta and in the carotid sinuses are instrumental in the genesis of HRV. The 20-minute supine resting period ensures that the baroreceptors have adapted to the change in blood pressure due to assuming the supine position. Throughout the 20-minute ECG, the subject is asked to breathe at a frequency of 15/min (0.25 Hz). For guidance of the exact respiratory rhythm at 0.25 Hz, the Leiden Respiratory Metronome, a visual metronome developed by H. v. d. Vooren and M. Santunione (©Leiden University Medical Center, Cardiology Department, used with permission by C. A. Swenne) is displayed on a computer screen easily visible for the subject. The purpose of the metronome guided respiration is to standardize the ECG recording as much as possible with respect to the influence of the respiratory rate on the determination of spectral parameters of the HRV. Thus, by ensuring a standard supine resting period of 20 minutes preceding the ECG recording and an equal respiratory rate for all subjects, the major physiological mechanisms influencing short term HRV are controlled for, and any differences in HRV found between subjects or groups of subjects can be attributed to other factors that are characteristic to the study subject (e. g. pathological processes, or influence of other risk factors on HRV).

After the ECG recording, a non-fasting venous blood sample is drawn under standardized conditions according to the KORA/MONICA protocol [48,55,62] with the subject remaining supine.

A transthoracic echocardiogram is performed on the GE Vingmed Ultrasound Vivid Five System or on the GE Vingmed System Five Performance with a FPA 2.5 MHz probe and Echo Pac Software version 6.3 (GE Medical Systems Ultrasound, GE Ultraschall Deutschland, Solingen) by a certified study physician following the protocol of the SHIP Study [49,63]. It collects information on parameters derived from M-Mode and Doppler standard echocardiographic techniques. Parameters to be studied include measurements required for the determination of left ventricular mass and systolic and diastolic function. Images and measurements of the echocardiographic examination are saved on optical disc and video to permit offline reading. The examination is concluded with an explication of the immediately available examination results (e. g., elevated blood pressure values, major electrocardiographic or echocardiographic abnormalities) for the study subject by the study physician. Table 3 gives an overview of the examination components and equipment.

**Table 3:** Examination components and equipment of the CARLA Study

**Examination components**	**Parameters**	**Instruments**
Anthropometry	Body weight, body height, waist- and hip circumference, body mass index	Digital scales (SECA 701), body height measuring system (SECA 220), tape measure
Blood pressure	Systolic and diastolic blood pressure at the arm and the ankle, heart rate	OMRON HEM-705CP automated oscillometric blood pressure measurement device
Electrocardiogram	Minnesota-Code (MEANS), HRV parameters, general de- and repolarisation parameters in the ECG (LEADS)	1. Welch Allyn Cardio Control Diagnostic Medical Workstation, MD Recorder, 600 Hz sampling rate (10-sec/20-min ECG) 2. mtm Multitechmed DMS 300-9 holter ECG recorder, 1024 Hz sampling rate
Echocardiogram	Measures of systolic and diastolic function, heart valves, ventricular dimensions	GE Vivid Five/System Five with FPA 2.5 MHz probe

For a random sub-sample of 200 subjects, a 24-hour ECG is recorded with the DMS 300-9 holter ECG recording system with a sampling rate of 1024 Hz (mtm Multitechmed, Hünfelden, Germany).

### Laboratory analyses

The non-fasting venous blood samples include the collection of serum, EDTA plasma, citrate plasma and EDTA full blood. All serum and plasma samples are centrifuged at 4°C in-house by specially trained study laboratory personnel. A small part of the serum and EDTA plasma is sent for analysis of total, HDL and LDL cholesterol, triglycerides, glucose, haemoglobin A1c (HbA1c), creatinine, high-sensitive C-reactive Protein (CRP) and n-terminal brain natriuretic peptide (NT-proBNP) to a single central laboratory at the University of Leipzig. All laboratory analyses – except for the determination of cytokines – are undertaken by the Institute of Laboratory Medicine, Clinical Chemistry and Molecular Diagnostics (ILM) at the Leipzig University Clinics. The laboratory has been accredited according to the accreditation norms ISO 15180 and ISO 17025. The cytokines interleukin-1 and 6 (IL-1, IL-6) and tumour necrosis factor-alpha (TNF-α) are determined at the research laboratory of the Department of Medicine III at the Martin-Luther-University Halle-Wittenberg. The major part of the specimens is stored at -80°C for future analysis. Citrate plasma is stabilized with 10% metaphosphoric acid (0.1 ml plasma : 0.9 ml MPA) before freezing for subsequent analyses of ascorbic acid (vitamine C). Serum total cholesterol is analyzed with the enzymatic colorimetric CHOD-PAP method at the Modular system (Roche Diagnostics, Mannheim, Germany) [64]. A homogenous enzymatic colorimetric test is used for the determination of serum HDL and LDL cholesterol at the Modular system [64,65]. Serum triglycerides are measured applying the colorimetric enzymatic GPO-PAP assay at the Modular system [64]. Casual serum glucose is measured on the Modular system with the hexokinase method. For the analysis of HbA1c in EDTA anticoagulated blood, the High Performance Liquid Chromatography (HPLC) method is used with the Variant II system (BIO-RAD, Munich, Germany). Creatinine is measured colorimetrically enzymatically on the Modular system [64]. High-sensitive C-reactive proteine (hsCRP) is determined with a turbidimetric immunologic reaction assay enhanced by the addition of latex on the Modular system [64,66]. NT-proBNP is measured using an electro-chemo-illuminescence assay by Roche Diagnostics on the Elecsys system (Roche Diagnostics, Mannheim, Germany) [67,68]. IL-1, IL-6 and TNF-α are measured using ELISA-techniques.

Genetic analyses including the haplotype mapping of specific gene variants (SNPs) using a pathway-oriented approach are planned. SNPs of the following signal and metabolic paths important for ageing will be analysed using blood stored at -80°C: 1.) the TNF-α/NF-κB pathway relevant for inflammatory factors and immune senescence; 2.) the IGF-1/PI3K/Akt longevity pathway; 3.) candidate genes for ageing (RAGE, ApoE, Klotho, interleukin-6 promotor polymorphism). Genes showing a positive association with a reduced HRV or a reduced cardiac function indicating cardiac ageing will be examined regarding their specific function and regulation in the context of cardiac ageing.

For a random subsample of 400 subjects, further laboratory parameters from frozen samples will be analysed, e. g. lipoprotein (a), vitamins, folic acid, homocysteine and thyroid hormones.

### ECG analyses

All 10-second ECGs are processed by the Modular ECG Analysis System (MEANS) [69] to obtain Minnesota Codes [70]. The MEANS program has been extensively validated [69,71-73]. MEANS also processes the 20-minute ECGs to obtain the locations and types of the QRS complexes. This information is then used to analyse HRV computing standard time domain and frequency domain parameters of HRV. HRV is computed according to the procedure used in previous studies. The method has been described elsewhere [47,74,75]. Briefly, artefacts and ectopic beats are replaced by interpolated normal sinus beats to a maximum of ten percent. For each ECG, the percentage of replaced beats is calculated. ECGs which display non-sinus rhythm, like atrial fibrillation, or where the majority of QRS complexes is paced by an artificial pacemaker, are excluded from HRV analysis.

Then, time domain parameters of HRV are calculated. The calculated time domain HRV parameters include the standard deviation of normal intervals (SDNN), the mean absolute successive normal interval differences, the root mean square successive normal interval differences (rMSSD), and the percentage of successive normal interval differences >50 ms (pNN50).

Prior to spectral analysis of the ECG, the tachogram has to undergo further processing steps. The further pre-analytic treatment of the inter-beat-interval (IBI) tachogram, which is the graphical presentation of RR intervals over time, includes an adjustment for linear trends, tachogram tapering and zero padding. A test of stationarity is performed, and non-stationary recordings are excluded from analysis. ECGs with extreme HRV values or high in-stationarity scores are visually checked to identify possible sources of error or explanations for the extreme values. If only part of the ECG is responsible for the lack of overall stationarity, stationary sub-sections of the 20-minute ECG are identified and selected for repeated HRV analysis.

For spectral density estimation of frequency domain variables, a fast Fourier transformation is employed to calculate very low frequency power (VLF power), low frequency power (LF power), high frequency power (HF power) and total power (TP).

Those HRV parameters are also calculated from 24-hr ECGs. The ranking of the subject regarding the distribution of HRV parameters in the study population and the diagnosis of a reduced HRV are compared between results derived from 20-minute ECGs and from 24-hour ECGs.

The addition of further ECG parameters – such as parameters describing T-wave complexity [76], the ventricular gradient, and the spatial angle between the QRS and T axes [77] – characterizing relevant physiological and pathophysiological processes which might improve the understanding of cardiovascular disease mechanisms – have been incorporated into the MEANS programme (MI-EUR) and into the LEADS programme (LUMC) [78] and are available for future analysis.

### Echocardiographic analyses

The transthoracic echocardiographic examinations are stored as digital files in order to permit off-line reading of the parameters independent of the examination procedure itself. However, for immediate documentation and interpretation by the study physician, online reading is performed during the examination. The analysis includes parameters of left ventricular dimension (left ventricular mass) and of systolic and diastolic function derived from M-Mode and Doppler echocardiographic measurements.

### Quality control – training and certification of study personnel and data quality management

In order to ensure a highly standardized data collection, all study personnel is specifically trained and certified for the study procedures. Interviewers and the study nurse were trained and certified in cooperation with the KORA study centre Augsburg before commencement of data collection in the study. Apart from certification examinations, a high level of standardization and examination quality is ensured by repeated supervisions of the study nurse and interviewers (by the principal investigator) during the examination or interview of study subjects.

For means of quality control, all interviews are digitally recorded, and a randomly selected sample of 10% of all interview recordings is monitored by comparing audiorecording with the data entry. In addition, interviews found to contain implausible answers during the computerized plausibility checks are monitored using the audiorecording.

The CARLA study physicians participate in the observer and reader certification for the echocardiographic examination held at the SHIP study centre Greifswald for echocardiographers of the SHIP and KORA study.

All paper documentation is double entered in order to minimize errors due to data entry. Visual and computerized plausibility checks are performed to detect possible data entry errors of paper documentation, examination procedures or interview. (The plausibility checks applied to ECG data have been described briefly above.)

### Statistical analysis

The statistical analysis includes the calculation of frequency distributions including means and medians of risk factors and HRV parameters by age, sex and cardiovascular disease status, the calculation of the prevalence of adverse risk factor levels and cardiovascular diseases, the calculation of correlation coefficients, and linear and logistic regression analyses. Differences between groups are tested using the t-test, the F-test or a test for trend. Standard errors, standard deviations and 95% confidence intervals are calculated to evaluate precision of the estimates. All statistical analyses are performed with SAS, Version 9 (SAS Institutes, Cary, NC). To assess validity of the 20-minute ECG compared to the gold standard (24-hour ECG), standard measures for diagnostic tests (sensitivity, specificity, positive and negative predictive value) are calculated.

### Sample size calculation

The sample size calculation was performed according to the primary outcome "Occurrence of reduced heart rate variability (RHRV)". This is to be estimated among subjects free from cardiac disease. We assumed that those make up about 50% in all age-sex strata. Assuming a true RHRV of 10%, a two-sided 95% confidence interval for this prevalence will have a one-sided length of 5.6% [4.4%; 15.6%] with 110 subjects free form cardiac disease. As such, a total of 220 subjects per 10-year-age-sex-stratum will be included, and a total sample size of 1760 subjects is needed for eight strata.

## Discussion

In the ageing populations of industrialized nations, the increasing burden of chronic cardiovascular diseases already has an enormous impact on population health, the health care system and the economy. The need for a better understanding of how to achieve "healthy ageing", how to slow down the processes of cardiovascular disease generation and progression, and how to improve preventive and therapeutic strategies is obvious in societies with a steadily rising life expectancy. But a better understanding of the disease processes is also essential for the reversal of the deleterious effect of the political and economic disruptions in Eastern Europe since the late 1980s on the population health with sharply increasing mortality and decreasing life expectancy. There is increasing evidence for an important contribution of the dysbalance of the autonomic nervous system with predominance of the sympathetic system to disease processes. There are conflicting results regarding a possible mediating role of autonomic dysfunction on the pathway from socioeconomic and psychosocial risk factors to cardiovascular disease, but population-based data on HRV are scarce [39-42,79,80].

The CARLA study aims to create a database for a detailed analysis of the association of markers of autonomic dysfunction with other cardiovascular risk factors on the path to cardiovascular disease. The wide age range of the present study population, being representative of a general population, permits the analysis of age-dependent effects and of processes which become more important at older age. Due to the selection of standardized examination procedures and interview items applied in other regional studies, this study serves for comparative analyses which might increase the chance to discover pathways responsible for the CVD epidemic in some populations. The recording of 20-minute ECGs in a large sample of the elderly general population gives the unique opportunity to examine markers of autonomic dysfunction with a higher precision than the shorter ECGs recorded in most other population-based studies to date. The additional comparison with 24-hour ECGs in the same study population increases the diagnostic security of the short-term ECGs. The study of further parameters of ECG physiology could create a deeper understanding of pathophysiologic processes responsible for the development and progression of cardiovascular diseases. This is a prerequisite of targeted preventive and therapeutic strategies needed in populations with a growing percentage of elderly persons with naturally high disease rates or in populations with increased numbers of premature cardiovascular disease.

## Conclusion

With the analysis of potential pathways from established and newer cardiovascular risk factors to cardiovascular disease, the CARLA study contributes important information needed for a successful change towards healthier ageing which can only be achieved by strengthening prevention as well as improving detection and supporting therapeutic steps in the management of cardiovascular diseases.

## List of abbreviations

ARIC = Atherosclerosis Risk in Communities

ATC codes = Anatomic Therapeutic Classification

BIPS = Bremen Institute for Prevention Research and Social Medicine

CARLA = **CAR**diovascular disease, **L**iving and **A**geing in Halle

CES-D = Center of Epidemiological Studies Depression Scale

CHD = Coronary Heart Disease

CHF = Chronic Heart Failure

CVD = Cardiovascular Disease

ECG = Electrocardiogram

EPIC = European Prospective Investigation into Cancer and Nutrition

GKV = Gesetzliche Krankenversicherung (Compulsory Health Insurance)

GSF = National Research Center for Environment and Health, Neuherberg, Germany

HAPIEE = Health, Alcohol and Psychosocial Factors in Eastern Europe

HRV = Heart rate variability

IDOM = computer-based system of medication recording using the official GKV medication database

IL-1, IL-6 = interleukin-1, interleukin-6

LEADS = Interactive research-oriented ECG/VCG analysis system

KORA = Cooperative Health Research in the Augsburg Region

MEANS = Modular ECG Analysis System

MONICA = Monitoring Trends and Determinants in Cardiovascular Disease

NT-proBPN = n-terminal pro Brain Natriuretic Peptide

PZN = central pharmanumber

SF 12 = Short Form Health Survey Questionnaire

SHIP = Study of Health in Pomerania

SNP = Single Nucleotide Polymorphism

TNF-α = tumornecrosis factor α

## Competing interests

The author(s) declare that they have no competing interests.

## Authors' contributions

KHG conceived of the study, designed major parts of the study, trained the study personnel, coordinates the study, participates in the statistical analyses and drafted the manuscript.

AK helps coordinate the study, participates in the statistical analyses and helped drafting the manuscript.

BS helped designing the interview, helps coordinating the study, participates in the statistical analyses and helped drafting the manuscript.

JAK helped drafting the manuscript and performs the Minnesota coding and pre-processing of ECGs for HRV analysis and contributed considerably to the MEANS software.

CAS helped designing the ECG recording procedure and drafting the manuscript, designed the HRV software and the LEADS software, and performs the HRV analyses.

OK helped drafting the manuscript, selecting the statistical procedures and participates in the statistical analyses.

JH helped designing the study and drafting the manuscript.

KW helped designing the study, coordinating the echocardiographic examinations, and drafting the manuscript.

## Pre-publication history

The pre-publication history for this paper can be accessed here:



## References

[B1] Association AH (2005). Heart Disease and Stroke. Statistics - 2005 Update.

[B2] Mosterd A, Hoes AW, Grobbee DE (1998). Epidemiology of heart failure: contours of an impending epidemic?. Neth J Med.

[B3] Mosterd A, Hoes AW, de Bruyne MC, Deckers JW, Linker DT, Hofman A, Grobbee DE (1999). Prevalence of heart failure and left ventricular dysfunction in the general population; The Rotterdam Study. Eur Heart J.

[B4] Raymond I, Pedersen F, Steensgaard-Hansen F, Green A, Busch-Sorensen M, Tuxen C, Appel J, Jacobsen J, Atar D, Hildebrandt P (2003). Prevalence of impaired left ventricular systolic function and heart failure in a middle aged and elderly urban population segment of Copenhagen. Heart.

[B5] Roger VL, Weston SA, Redfield MM, Hellermann-Homan JP, Killian J, Yawn BP, Jacobsen SJ (2004). Trends in heart failure incidence and survival in a community-based population. JAMA.

[B6] Haldeman GA, Croft JB, Giles WH, Rashidee A (1999). Hospitalization of patients with heart failure: National Hospital Discharge Survey, 1985 to 1995. Am Heart J.

[B7] O'Connell JB (2000). The economic burden of heart failure. Clin Cardiol.

[B8] O'Connor CM, Friesinger GC, Topol E (1998). Aging and the heart. Textbook of cardiovascular medicine.

[B9] Stewart S, MacIntyre K, Capewell S, McMurray JJ (2003). Heart failure and the aging population: an increasing burden in the 21st century?. Heart.

[B10] Bobak M, Marmot M (1996). East-West mortality divide and its potential explanations: proposed research agenda. BMJ.

[B11] Walberg P, McKee M, Shkolnikov V, Chenet L, Leon DA (1998). Economic change, crime, and mortality crisis in Russia: regional analysis. BMJ.

[B12] WHO (1994). Ecological analysis of the association between mortality and major risk factors of cardiovascular disease. The World Health Organization MONICA Project. Int J Epidemiol.

[B13] Willich S, Löwel H, Mey W, Trautner C (1999). Regional Variations in Mortality of Cardiovascular Diseases in Germany.. Deutsches Ärzteblatt.

[B14] Hemingway H, Marmot M, al. YS (1998). Psychosocial factors in the primary and secondary prevention of coronary heart disease: a systematic review.. Evidence based cardiology.

[B15] Kristenson M, Orth-Gomer K, Kuchinskiene Z, Hertzman C and al  (1996). Different patterns of psychosocial strain; a possible explanation for the differences in ischemic heart disease mortality between Sweden and Lithuania?. East-West Life Expectancy Gap in Europe.

[B16] Peter R, Alfredsson L, Hammar N, Siegrist J, Theorell T, Westerholm P (1998). High effort, low reward, and cardiovascular risk factors in employed Swedish men and women: baseline results from the WOLF Study. J Epidemiol Community Health.

[B17] Ferketich AK, Schwartzbaum JA, Frid DJ, Moeschberger ML (2000). Depression as an antecedent to heart disease among women and men in the NHANES I study. National Health and Nutrition Examination Survey. Arch Intern Med.

[B18] Stansfeld SA, Fuhrer R, Shipley MJ, Marmot MG (2002). Psychological distress as a risk factor for coronary heart disease in the Whitehall II Study. Int J Epidemiol.

[B19] Rosengren A, Hawken S, Ounpuu S, Sliwa K, Zubaid M, Almahmeed WA, Blackett KN, Sitthi-amorn C, Sato H, Yusuf S (2004). Association of psychosocial risk factors with risk of acute myocardial infarction in 11119 cases and 13648 controls from 52 countries (the INTERHEART study): case-control study. Lancet.

[B20] Marmot MG, Smith GD, Stansfeld S, Patel C, North F, Head J, White I, Brunner E, Feeney A (1991). Health inequalities among British civil servants: the Whitehall II study. Lancet.

[B21] Marmot MG, Bosma H, Hemingway H, Brunner E, Stansfeld S (1997). Contribution of job control and other risk factors to social variations in coronary heart disease incidence. Lancet.

[B22] Labarthe DR (1998). Adverse Psychosocial Patterns. Epidemiology and Prevention of Cardiovascular Diseases A Global Challenge.

[B23] Labarthe DR (1998). Social Conditions. Epidemiology and Prevention of Cardiovascular Diseases A Global Challenge.

[B24] Liao D, Barnes RW, Chambless LE, Simpson RJJ, Sorlie P, Heiss G (1995). Age, race, and sex differences in autonomic cardiac function measured by spectral analysis of heart rate variability--the ARIC study. Atherosclerosis Risk in Communities. Am J Cardiol.

[B25] Agelink MW, Malessa R, Baumann B, Majewski T, Akila F, Zeit T, Ziegler D (2001). Standardized tests of heart rate variability: normal ranges obtained from 309 healthy humans, and effects of age, gender, and heart rate. Clin Auton Res.

[B26] Tsuji H, Venditti FJJ, Manders ES, Evans JC, Larson MG, Feldman CL, Levy D (1994). Reduced heart rate variability and mortality risk in an elderly cohort. The Framingham Heart Study. Circulation.

[B27] Liao D, Cai J, Rosamond WD, Barnes RW, Hutchinson RG, Whitsel EA, Rautaharju P, Heiss G (1997). Cardiac autonomic function and incident coronary heart disease: a population-based case-cohort study. The ARIC Study. Atherosclerosis Risk in Communities Study. Am J Epidemiol.

[B28] Huikuri HV, Makikallio TH, Airaksinen KE, Seppanen T, Puukka P, Raiha IJ, Sourander LB (1998). Power-law relationship of heart rate variability as a predictor of mortality in the elderly. Circulation.

[B29] de Bruyne MC, Kors JA, Hoes AW, Klootwijk P, Dekker JM, Hofman A, van Bemmel JH, Grobbee DE (1999). Both decreased and increased heart rate variability on the standard 10-second electrocardiogram predict cardiac mortality in the elderly: the Rotterdam Study. Am J Epidemiol.

[B30] Dekker JM, Crow RS, Folsom AR, Hannan PJ, Liao D, Swenne CA, Schouten EG (2000). Low heart rate variability in a 2-minute rhythm strip predicts risk of coronary heart disease and mortality from several causes: the ARIC Study. Atherosclerosis Risk In Communities. Circulation.

[B31] Bigger JT, Fleiss JL, Rolnitzky LM, Steinman RC (1993). The ability of several short-term measures of RR variability to predict mortality after myocardial infarction. Circulation.

[B32] Tsuji H, Larson MG, Venditti FJJ, Manders ES, Evans JC, Feldman CL, Levy D (1996). Impact of reduced heart rate variability on risk for cardiac events. The Framingham Heart Study. Circulation.

[B33] Nolan J, Batin PD, Andrews R, Lindsay SJ, Brooksby P, Mullen M, Baig W, Flapan AD, Cowley A, Prescott RJ, Neilson JM, Fox KA (1998). Prospective study of heart rate variability and mortality in chronic heart failure: results of the United Kingdom heart failure evaluation and assessment of risk trial (UK-heart). Circulation.

[B34] Janszky I, Ericson M, Mittleman MA, Wamala S, Al Khalili F, Schenck-Gustafsson K, Orth-Gomer K (2004). Heart rate variability in long-term risk assessment in middle-aged women with coronary heart disease: The Stockholm Female Coronary Risk Study. J Intern Med.

[B35] Horsten M, Ericson M, Perski A, Wamala SP, Schenck-Gustafsson K, Orth-Gomer K (1999). Psychosocial factors and heart rate variability in healthy women. Psychosom Med.

[B36] Carney RM, Blumenthal JA, Stein PK, Watkins L, Catellier D, Berkman LF, Czajkowski SM, O'Connor C, Stone PH, Freedland KE (2001). Depression, heart rate variability, and acute myocardial infarction. Circulation.

[B37] Sloan RP, Bagiella E, Shapiro PA, Kuhl JP, Chernikhova D, Berg J, Myers MM (2001). Hostility, gender, and cardiac autonomic control. Psychosom Med.

[B38] Virtanen R, Jula A, Salminen JK, Voipio-Pulkki LM, Helenius H, Kuusela T, Airaksinen J (2003). Anxiety and hostility are associated with reduced baroreflex sensitivity and increased beat-to-beat blood pressure variability. Psychosom Med.

[B39] Carney RM, Blumenthal JA, Freedland KE, Stein PK, Howells WB, Berkman LF, Watkins LL, Czajkowski SM, Hayano J, Domitrovich PP, Jaffe AS (2005). Low heart rate variability and the effect of depression on post-myocardial infarction mortality. Arch Intern Med.

[B40] Hemingway H, Shipley M, Brunner E, Britton A, Malik M, Marmot M (2005). Does autonomic function link social position to coronary risk? The Whitehall II study. Circulation.

[B41] Janszky I, Ericson M, Blom M, Georgiades A, Magnusson JO, Alinagizadeh H, Ahnve S (2005). Wine drinking is associated with increased heart rate variability in women with coronary heart disease. Heart.

[B42] Steptoe A, Feldman PJ, Kunz S, Owen N, Willemsen G, Marmot M (2002). Stress responsivity and socioeconomic status: a mechanism for increased cardiovascular disease risk?. Eur Heart J.

[B43] Vasan RS, Sullivan LM, Roubenoff R, Dinarello CA, Harris T, Benjamin EJ, Sawyer DB, Levy D, Wilson PW, D'Agostino RB (2003). Inflammatory markers and risk of heart failure in elderly subjects without prior myocardial infarction: the Framingham Heart Study. Circulation.

[B44] Ingelsson E, Arnlov J, Sundstrom J, Lind L (2005). Inflammation, as measured by the erythrocyte sedimentation rate, is an independent predictor for the development of heart failure. J Am Coll Cardiol.

[B45] Sajadieh A, Nielsen OW, Rasmussen V, Hein HO, Abedini S, Hansen JF (2004). Increased heart rate and reduced heart-rate variability are associated with subclinical inflammation in middle-aged and elderly subjects with no apparent heart disease. Eur Heart J.

[B46] (1996). Heart rate variability. Standards of measurement, physiological interpretation, and clinical use. Task Force of the European Society of Cardiology and the North American Society of Pacing and Electrophysiology. Eur Heart J.

[B47] Dekker JM, De Vries EL, Lengton RR, Maan AC, Schouten EG, Swenne CA, Maan A (1996). Reproducibility and comparability of short- and long-term heart rate variability measures in healthy young men.. Ann Noninvasive Electrocardiol.

[B48] Hense HW, Filipiak B, Döring A, Stieber J, Liese A, Keil U (1998). Ten-year trends of cardiovascular risk factors in the MONICA Augsburg Region in Southern Germany. Results from the 1984/85, 1989/90 and 1994/1995 surveys.. CVD Prevention.

[B49] John U, Greiner B, Hensel E, Ludemann J, Piek M, Sauer S, Adam C, Born G, Alte D, Greiser E, Haertel U, Hense HW, Haerting J, Willich S, Kessler C (2001). Study of Health In Pomerania (SHIP): a health examination survey in an east German region: objectives and design. Soz Praventivmed.

[B50] Kroke A, Klipstein-Grobusch K, Voss S, Moseneder J, Thielecke F, Noack R, Boeing H (1999). Validation of a self-administered food-frequency questionnaire administered in the European Prospective Investigation into Cancer and Nutrition (EPIC) Study: comparison of energy, protein, and macronutrient intakes estimated with the doubly labeled water, urinary nitrogen, and repeated 24-h dietary recall methods. Am J Clin Nutr.

[B51] Hofman A, Grobbee DE, de Jong PT, van den Ouweland FA (1991). Determinants of disease and disability in the elderly: the Rotterdam Elderly Study. Eur J Epidemiol.

[B52] Bobak M, Room R, Pikhart H, Kubinova R, Malyutina S, Pajak A, Kurilovitch S, Topor R, Nikitin Y, Marmot M (2004). Contribution of drinking patterns to differences in rates of alcohol related problems between three urban populations. J Epidemiol Community Health.

[B53] Giesecke B, Nagl H, GmbH GSFFUG (2001). DAIMON -Rechnergestützte Fragebogenentwicklung und Interviewführung. Dokumentation. GSF-Bericht 03/01..

[B54] Rose G, McCartney P, Reid DD (1977). Self-administration of a questionnaire on chest pain and intermittent claudication. Br J Prev Soc Med.

[B55] Döring A, Fischer B, Holle R, Hoppe S, Immervoll T, Janssen C, John J, Merkl J, Nagl H, Mühlberger N, Papke K, Perz S, Pietsch M, Rathmann W, Schäfer T, Schwertner B, Stieber J, Zahlmann G (2000). KORA-Survey 2000. Manual of Operation. Untersucher-Handbuch..

[B56] Fricke U, Günther J, Zawinell A (2005). Anatomisch-therapeutisch-chemische Klassifikation mit Tagesdosen für den deutschen Arzneimittelmarkt. Methodik der ATC-Klassifikation und DDD-festlegung. ATC-Index mit DDD-Angaben. GKV-Arzneimittelindex.

[B57] Siegrist J (1996). Adverse health effects of high-effort/low-reward conditions. J Occup Health Psychol.

[B58] Siegrist J, Starke D, Chandola T, Godin I, Marmot M, Niedhammer I, Peter R (2004). The measurement of effort-reward imbalance at work: European comparisons. Soc Sci Med.

[B59] O'Brien E, Mee F, Atkins N, Thomas M (1996). Evaluation of three devices for self-measurement of blood pressure according to the revised British Hypertension Society Protocol: the Omron HEM-705CP, Philips HP5332, and Nissei DS-175. Blood Press Monit.

[B60] Mundt KA, Chambless LE, Burnham CB, Heiss G (1992). Measuring ankle systolic blood pressure: validation of the Dinamap 1846 SX. Angiology.

[B61] National Heart Lung and Blood Institute of the National Institutes of Health (1987). ARIC Manuals of Operation. Manual 5 Electrocardiography.

[B62] (1990). WHO MONICA Project. The MONICA Manual..

[B63] Piek M, Lüdeman J, Völzke H, Hummel A, Hense HW (2002). Operationshandbuch Echokardiographie. Study of Health in Pomerania (SHIP-I). 1. Follow-up-Untersuchung.

[B64] Thomas L (2005). Labor und Diagnose.

[B65] Kimberly MM, Leary ET, Cole TG, Waymack PP (1999). Selection, validation, standardization, and performance of a designated comparison method for HDL-cholesterol for use in the cholesterol reference method laboratory network. Clin Chem.

[B66] Eda S, Kaufmann J, Roos W, Pohl S (1998). Development of a new microparticle-enhanced turbidimetric assay for C-reactive protein with superior features in analytical sensitivity and dynamic range. J Clin Lab Anal.

[B67] Richards AM, Nicholls MG, Yandle TG, Frampton C, Espiner EA, Turner JG, Buttimore RC, Lainchbury JG, Elliott JM, Ikram H, Crozier IG, Smyth DW (1998). Plasma N-terminal pro-brain natriuretic peptide and adrenomedullin: new neurohormonal predictors of left ventricular function and prognosis after myocardial infarction. Circulation.

[B68] Struthers AD (1999). How to use natriuretic peptide levels for diagnosis and prognosis. Eur Heart J.

[B69] van Bemmel JH, Kors JA, van Herpen G (1990). Methodology of the modular ECG analysis system MEANS. Methods Inf Med.

[B70] Prineas RJ, Crow. RS, Blackburn H (1982). The Minnesota Code Manual of Electrocardiographic Findings Standard Procedures for Measurement and Classification.

[B71] Kors JA, van Herpen G, Wu J, Zhang Z, Prineas RJ, van Bemmel JH (1996). Validation of a new computer program for Minnesota coding. J Electrocardiol.

[B72] de Bruyne MC, Kors JA, Hoes AW, Kruijssen DA, Deckers JW, Grosfeld M, van Herpen G, Grobbee DE, van Bemmel JH (1997). Diagnostic interpretation of electrocardiograms in population-based research: computer program research physicians, or cardiologists?. J Clin Epidemiol.

[B73] Kors JA, Crow RS, Hannan PJ, Rautaharju PM, Folsom AR (2000). Comparison of computer-assigned Minnesota Codes with the visual standard method for new coronary heart disease events. Am J Epidemiol.

[B74] Bootsma M, Swenne CA, Van Bolhuis HH, Chang PC, Cats VM, Bruschke AV (1994). Heart rate and heart rate variability as indexes of sympathovagal balance. Am J Physiol.

[B75] Pluim BM, Swenne CA, Zwinderman AH, Maan AC, van der LA, Doornbos J, Van der Wall EE (1999). Correlation of heart rate variability with cardiac functional and metabolic variables in cyclists with training induced left ventricular hypertrophy. Heart.

[B76] Priori SG, Mortara DW, Napolitano C, Diehl L, Paganini V, Cantu F, Cantu G, Schwartz PJ (1997). Evaluation of the spatial aspects of T-wave complexity in the long-QT syndrome. Circulation.

[B77] Mirvis DM, Goldberger AL, Zipes DP, Libby P, Bonow RO and Braunwald E (2005). Electrocardiography. Braunwald's Heart Disease.

[B78] Draisma HHM, Swenne CA, Van de Vooren H, Maan AC, Hooft van huysduynen B, Van der Wall EE, Schalij MJ (2005). LEADS, an interactive research-oriented ECG/VCG analysis system. Computers in Cardiology.

[B79] Gallo LC, Bogart LM, Vranceanu AM, Walt LC (2004). Job characteristics, occupational status, and ambulatory cardiovascular activity in women. Ann Behav Med.

[B80] Riese H, Van Doornen LJ, Houtman IL, De Geus EJ (2004). Job strain in relation to ambulatory blood pressure, heart rate, and heart rate variability among female nurses. Scand J Work Environ Health.

[B81] Jöckel KH, Babitsch B, Bellach BM, Bloomfield K, Hoffmeyer-Zlotnik J, Winkler J, Wolf C, Ahrens W, Bellach BM and Jöckel KH (1998). Messung und Quantifizierung soziodemographischer Merkmale in epidemiologischen Studien. Messung soziodemographischer Merkmale in der Epidemiologie.

[B82] Adam C, Alte D, Born G, Eichenauer-Rettig U, John U, Lüdemann J, Paritschke H, Piek M, Sauer S, Wussow T, Härtel U (2001). Operationshandbuch Computergestütztes Interview. Study of Health in Pomerania (SHIP-I). 1. Follow-up-Untersuchung.

[B83] Keil U, Cairns V, Döring A, Härtel U, Jorcik J, Perz S, Stieber J (1985). MONICA-Augsburg Manual of Operations - Survey. GSF-Bericht 20/85.

[B84] (1988). The World Health Organization MONICA Project (monitoring trends and determinants in cardiovascular disease): a major international collaboration. WHO MONICA Project Principal Investigators. J Clin Epidemiol.

[B85] Bothig S (1989). WHO MONICA Project: objectives and design. Int J Epidemiol.

[B86] Winkler G, Döring A (1995). Kurzmethoden zur Charakterisierung des Ernährungsmusters: Einsatz und Auswertung eines Food-Frequency-Fragebogens. Ernährungs-Umschau.

[B87] Baecke JA, Burema J, Frijters JE (1982). A short questionnaire for the measurement of habitual physical activity in epidemiological studies. Am J Clin Nutr.

[B88] Berkman LF, Syme SL (1979). Social networks, host resistance, and mortality: a nine-year follow-up study of Alameda County residents. Am J Epidemiol.

[B89] Seeman TE, Berkman LF (1988). Structural characteristics of social networks and their relationship with social support in the elderly: who provides support. Soc Sci Med.

[B90] Putnam RD, Leonardi R, Nanetti RY (1993). Making democracy work: Civic traditions in modern Italy.

[B91] Rose R (1996). New Russia Barometer VI: After the presidential election..

[B92] Rose R (2001). How free from fear are citizen in transition societies?. http://www.worldbank.org/transitionnewsletter/mayjune2002/pgs18-20.htm.

[B93] Bullinger M, Kirchberger I (1998). SF-36 Fragebogen zum Gesundheitszustand (Handanweisung).

[B94] Hautzinger M (1988). Die CES-D Skala. Ein Depressionsmessinstrument für Untersuchungen in der Allgemeinbevölkerung.. Diagnostika.

[B95] Hautzinger M, Bailer M (1996). Allgemeine Depressionsskala (ADS).

[B96] Kohlmann TH, Gerbershagen HU (2005). CES-D, Deutsche Version.

